# Empowering and including ‘seldom heard’ communities in systems thinking for weight management

**DOI:** 10.1177/17579139231180732

**Published:** 2023-11-27

**Authors:** L Nield

**Affiliations:** Advanced Wellbeing Research Centre, Sheffield Hallam University, Olympic Legacy Park, Sheffield S9 3TU, UK

*This article initiates an important conversation about how underrepresentation of stakeholders risks perpetuating health inequalities by designing seldom-heard communities out of the system*.

Obesity research, service provision and policy have attempted to stem the tide of obesity to alleviate financial, social and healthcare pressures. While much of this work has been well-intentioned, well-designed and well-managed, outcomes for weight loss are poor, and weight regain is common.^1^ The prevalence of obesity is associated with deprivation, gender, ethnicity, household income and geographic location,^2^ confirming that obesity is a disease of inequality.

Weight management is recognised to be complex as highlighted by the Foresight obesity systems map which challenged the simple ‘energy in vs. energy out’ rhetoric.^3^ In recognition of the complexity of factors at play, attention has turned to a whole systems approach (WSA) to address such complex issues.^4^

A system is defined as ‘a set of inter-connected parts that have to function together to be effective’.^5^ There is no single agreed definition of a health system, and as such, healthcare and public health are often described in academic literature as separate systems.^6^ The health system is therefore separate from, but influenced by, larger systems including political and social systems.^6^ Within a traditional biomedical-focused health system, ‘health’ may be attributed to individual factors including access to and participation in public health and healthcare services. However, the wider determinants of health recognise the significant influence of sociocultural, economic, environmental and political factors on health.^7^

The Institute of Health Equity report (2018) proposed a broad health system approach to improve and tackle health inequalities and advocated for a place-based health system which focuses on prevention and treatment of ill-health, understands local population health risks, collaborates across sectors, acts on social determinants of health and develops ‘proportionate universalist’ approaches.^8^ Despite this, weight management policy and provision has not adequately addressed the multifaceted causes of obesity and continues to focus on individual behaviour change approaches putting the onus for weight loss on individuals, with success or failure dependent on their personal agency.^9^

**Figure 1.**
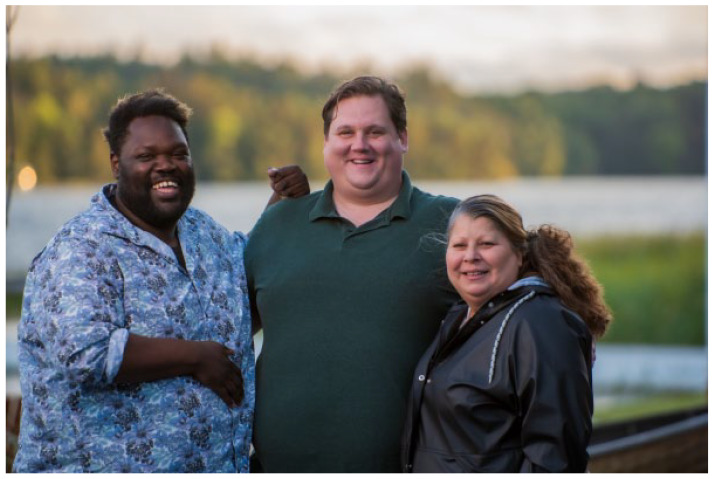
credit: ecpomedia.org

Population health approaches drive public health outcomes and are key to systems thinking. Population health extends beyond the health system and is based on an ecological model of health, considering how individual, social and environmental determinants influence health and recognising^10^ that people are active participants in their own health outcomes. It is, therefore, important to recognise that individual health and health outcomes are underpinned by both public health and healthcare activities and also by how individuals are enabled to interact with these systems and their broader social environments.^6^

The 2010 Marmot review highlighted the structural inequalities driven by the social determinants of health and argued for change to prevent ill-health and social injustice caused by inequality and to protect the health and wellbeing of future generations. It described how inequalities across communities are driven by inequalities in health and clearly articulated the need for community empowerment to reduce health inequalities.^11^

Many of the factors which prevent engagement with and adherence to current weight management services demonstrate that such interventions^12^ are inappropriate for individuals from underserved and more deprived groups, and as a result, lack of engagement with these populations continues to drive health inequality. It highlights the need for a significant overhaul of current weight management provision, embracing a more systems-led approach and for the voices of underserved and seldom heard communities to be involved in the design and development of weight management provision. Participatory methodologies such as co-design and co-production are crucial to systems approaches and understanding the needs and demands of these underserved groups in a considerate rather than tokenistic way.^13^

The inclusion of stakeholder networks is vital.^14^ In the case of obesity, stakeholders should be representative of healthcare, actors within the wider system, and should also include users or potential users and beneficiaries of the system such as those living with or at risk of obesity.^15^ Each stakeholder may have a different viewpoint which allows a broader perspective and new insights into how the system works, what the problems are and why, what can be improved or changed, and the impact of changes on other components in the system.^16^ It is important that stakeholders are representative of the community and populations targeted by weight management systems. A recent systematic review concluded that the most successful WSA weight management and public health projects included effective community involvement where participants identified the needs and actively participated in solutions at a local level.^17^ The review also highlighted that whole systems thinking is in its infancy and is not consistently embedded into the implementation or evaluation of interventions. This is exemplified with few published studies successfully targeting ‘at risk’ population groups, such as low socioeconomic status, those with low educational attainment levels, and Black and minority ethnic groups.^17^ Not only does this restrict the usefulness of the findings but it also demonstrates how systems thinking in weight management has not always been inclusive and has engaged minimally with some communities, rendering them ‘seldom heard’.^15,17^

The term ‘seldom heard’ refers to under-represented communities, groups, populations or people who use or will potentially use services but who are less likely to be heard by professionals and decision-makers.^15^ However, the importance of including seldom heard groups in health and social care research is crucial on scientific, policy and ethical grounds.^18^ The under-representation of these groups in health research impacts the validity and generalisability of data,^19^ the development of services and interventions that meet their needs,^20^ allocation and access to resources^21^ and can perpetuate health inequalities, especially as some of these groups have more health needs.^22^

WSA success metrics have been proposed by the Public Health England (PHE) logic model which describes outcomes including a reduction in obesity levels and health inequalities, effective use of community and other assets and an overall improvement in population health and wellbeing.^4^ While the move towards, and expansion of systems thinking is encouraged, this model lacks patient-led outcomes and an understanding of ‘what matters most’ to populations involved in, and targeted by, weight management systems.

It is, therefore, of paramount importance that future obesity approaches adopt a strong WSA that is inclusive of the voices of underserved communities and that actively recruits and engages people from seldom heard groups in the identification of systemic issues, challenges and barriers, service design, delivery and development, and the implementation of actions for systems change and evaluation. Co-production and co-development methodologies need to be embedded within WSA from the start, and effort needs to be made to ensure that the participants are truly representative of the target populations. Without capturing the voices of these communities, WSA to weight management (including weight management provision) may inadvertently ignore the needs of those at high risk of obesity and perpetuate further health inequalities.
